# The *PLOS Computational Biology* Software Section

**DOI:** 10.1371/journal.pcbi.1002799

**Published:** 2012-11-29

**Authors:** Andreas Prlić, Hilmar Lapp

**Affiliations:** 1 Department of Pharmacology, University of California San Diego, La Jolla, California, United States of America; 2 National Evolutionary Synthesis Center (NESCent), Durham, North Carolina, United States of America

As a member of the PLOS family, *PLOS Computational Biology* promotes open and unrestricted access to scientific publications and the research products that support them. For *PLOS Computational Biology*, this specifically includes computational methods and the software that implements them. To foster a culture of open exchange and reuse of software, we created a new category of manuscripts called Software Articles. We have been accepting submissions in this category for over a year now, and all articles published in this category are now also available as an online collection (http://www.ploscollections.org/software).

This is a collection of articles spanning a wide range of different topics in computational biology, starting from video image analysis in “Automated Tracking of Whiskers in Videos of Head Fixed Rodents” [1], over to simulation and analysis of biological system models in “Hybrid Models and Biological Model Reduction with PyDSTool” [2]. We have published articles describing genomic tools, such as “Podbat: Genomic Tool for Epigenetic Meta-Analysis” [3] and “Exploring Massive, Genome Scale Datasets with the GenometriCorr Package” [4], computational chemistry, in “AutoClickChem: Click Chemistry *in Silico*” [5], and protein science, in “ProteinHistorian: Tools for the Comparative Analysis of Eukaryote Protein Origin” [6] and “CAVER 3.0: A Tool for the Analysis of Transport Pathways in Dynamic Protein Structures” [7]. As new Software Articles get published in this journal, they will be added to this collection.

## Why Do We Support Open-Source Scientific Software?

The benefits of openness in conducting and disseminating science are particularly evident in the development and publication of software, as suggested by the fact that open-source software principles are often cited as inspiration for advocating similar principles for science as a whole under the banner of open science. These benefits can be summarized as the following:

– **Reproducibility** for experiments, results, and data, due to the transparent and verifiable nature of the source code.
– **Faster development** of scientific applications, by being able to focus on solving research-specific problems while reusing existing tried-and-proven code for common, often mundane tasks, such as parsing files. Reusable open-source libraries for these, and even for reference implementations of widely used algorithms, exist, often in different programming languages. They allow solutions for problems to be used in different contexts than might have been anticipated by their original authors.
– **Increased quality** is a natural consequence of more eyes looking at the source code, and more developers trying to reuse the code in their own applications. Code that is being reused receives more users who apply the code to a wider range of inputs, which in return helps uncover inappropriate assumptions made during development and other critical issues. Finally, we all tend to be more careful in our work if we know it will be publicly inspected, and developers are no different.
– **Long-term availability**. As anyone who has ever tried to obtain software for an algorithm published some time ago will know, scientific software often becomes unavailable only a few years after its publication, unless the authors had the foresight to use an open-source repository for their development. This is one of the reasons why *PLOS Computational Biology* requires source code to be uploaded during submission of a manuscript, so that a copy of record can be preserved.

Besides these obvious benefits of open source software, another reason for creating the Software Section at *PLOS Computational Biology* was to highlight the value that software brings to the scientific endeavor. Software and those who develop it are generally underrated by the academic system. This is our effort to bring more recognition to them.

## What Are the Criteria for Publication in *PLOS Computational Biology*?

The idea of free reuse, redistribution, and modification of scientific software is at the core of the *PLOS Computational Biology* Software Section. For a manuscript to be published as a Software Article, we require that all software use a license approved by the Open Source Initiative (OSI). OSI's approval criteria (http://www.opensource.org/docs/osd) ensure transparency, reproducibility, and if applied to scientific software, push science forward by allowing researchers to build on existing work. Scientifically, we require that the article **has been shown to provide new biological insights** and are not only just incremental improvements over existing approaches. All articles require a **presubmission inquiry**. For a detailed description of all criteria see http://www.ploscompbiol.org/static/guidelines.action#software.

We look forward to submissions of more manuscripts to the Software Section and we hope that with this initiative we can further improve the quality and acceptance of open source in the scientific community.
